# *rac*-(1*S*,2*R*)-Diethyl 6-hydr­oxy-1-(4-methoxy­phen­yl)-3-oxo-2,3-di­hydro-1*H*-benzo[*f*]chromen-2-yl]­phospho­nate

**DOI:** 10.1107/S1600536808015857

**Published:** 2008-06-07

**Authors:** Jakub Wojciechowski, Henryk Krawczyk, Łukasz Albrecht, Wojciech M. Wolf

**Affiliations:** a Institute of General and Ecological Chemistry, Technical University of Łódź, ul. Żeromskiego 116, 90-924 Łódź, Poland; b Institute of Organic Chemistry, Technical University of Łódź, ul. Żeromskiego 116, 90-924 Łódź, Poland

## Abstract

In the title compound, C_24_H_25_O_7_P, the δ-valerolactonyl ring exists in a distorted screw-boat conformation with the diethoxy­phosphoryl substituent occupying an axial position. The latter adopts an almost *syn*-periplanar conformation around the P—C bond. The mol­ecules form centrosymmetric dimers connected by O—H⋯O hydrogen bonds.

## Related literature

For the biological activity of 4-aryl-3,4-dihydro­coumarins, see: Bailly *et al.* (2003 ▶); Roelens *et al.* (2005 ▶); Zhang *et al.* (2006 ▶). For their synthesis, see: Aoki *et al.* (2005 ▶); Krawczyk *et al.* (2007*a* ▶); Li *et al.* (2005 ▶); Rizzi *et al.* (2006 ▶). For a comparison structure, see: Krawczyk *et al.* (2007*b* ▶).

For the atomic charges fitted to electrostatic potential, see: Frisch *et al.* (2004 ▶); Breneman & Wiberg (1990 ▶). For repulsive interactions between O atoms, see: Gillespie & Popelier, (2001 ▶). For hydrogen-bond graph-set terminology, see: Bernstein *et al.* (1995 ▶); Etter (1990 ▶). For ring puckering analysis, see: Boeyens (1978 ▶); Cremer & Pople (1975 ▶); Frisch *et al.* (2004 ▶). For details of the Cambridge Structural Database, see: Allen (2002 ▶).
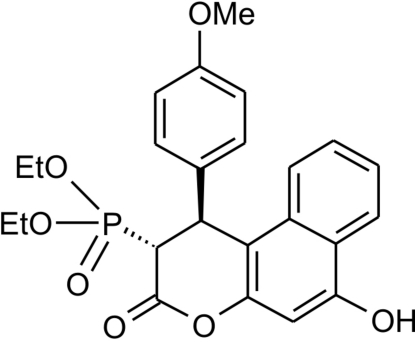


## Experimental

### 

#### Crystal data

C_24_H_25_O_7_P
*M* *_r_* = 456.41
Monoclinic, 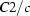

*a* = 21.6231 (17) Å
*b* = 10.0018 (8) Å
*c* = 22.4011 (17) Å
β = 111.806 (1)°
*V* = 4498.0 (6) Å^3^
*Z* = 8
Mo *K*α radiation
μ = 0.17 mm^−1^
*T* = 293 (2) K
0.30 × 0.20 × 0.15 mm

#### Data collection

Bruker SMART APEX diffractometer
Absorption correction: multi-scan (*SADABS*; Bruker, 2003 ▶) *T*_min_ = 0.951, *T*_max_ = 0.976
37601 measured reflections
5076 independent reflections
3585 reflections with *I* > 2σ(*I*)
*R*_int_ = 0.043

#### Refinement

*R*[*F* ^2^ > 2σ(*F* ^2^)] = 0.045
*wR*(*F* ^2^) = 0.137
*S* = 1.05
5076 reflections
310 parameters
H-atom parameters constrained
Δρ_max_ = 0.30 e Å^−3^
Δρ_min_ = −0.36 e Å^−3^

### 

Data collection: *SMART* (Bruker, 2003 ▶); cell refinement: *SAINT-Plus* (Bruker, 2003 ▶); data reduction: *SAINT-Plus*; program(s) used to solve structure: *SHELXS97* (Sheldrick, 2008 ▶); program(s) used to refine structure: *SHELXL97* (Sheldrick, 2008 ▶); molecular graphics: *SHELXTL* (Sheldrick, 2008 ▶); software used to prepare material for publication: *SHELXTL* and *publCIF* (Westrip, 2008 ▶).

## Supplementary Material

Crystal structure: contains datablocks global, I. DOI: 10.1107/S1600536808015857/ng2458sup1.cif

Structure factors: contains datablocks I. DOI: 10.1107/S1600536808015857/ng2458Isup2.hkl

Additional supplementary materials:  crystallographic information; 3D view; checkCIF report

**Fig. 4.**
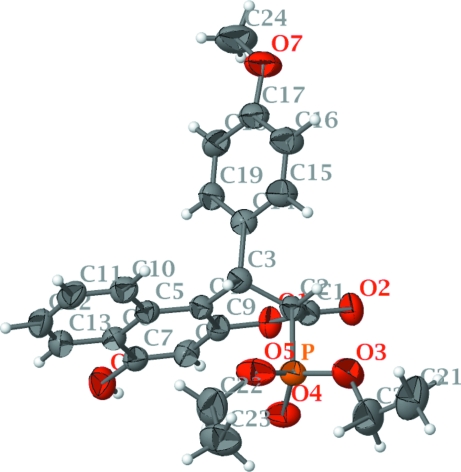
Supplementary figure.

Enhanced figure: interactive version of Fig. 4

## Figures and Tables

**Table 1:** Hydrogen-bond geometry (Å, °)

*D*—H⋯*A*	*D*—H	H⋯*A*	*D*⋯*A*	*D*—H⋯*A*
O6—H6⋯O4^i^	0.83	1.87	2.701 (2)	178

Symmetry code: (i) 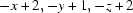.

**Fig. 1.**
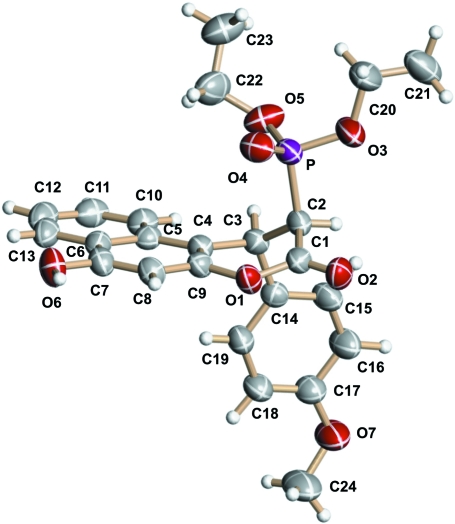
The molecule of the title compound (I). Displacement elipsoids are drawn at the 50% probability level.

**Fig. 2.**
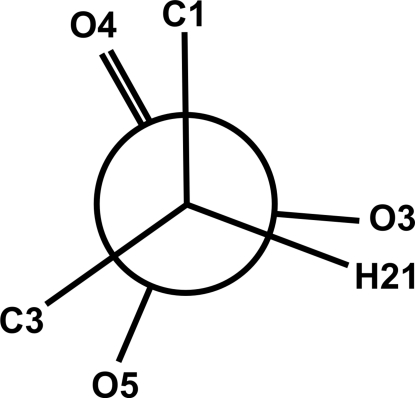
Newman projection along the C2—P bond.

**Fig. 3.**
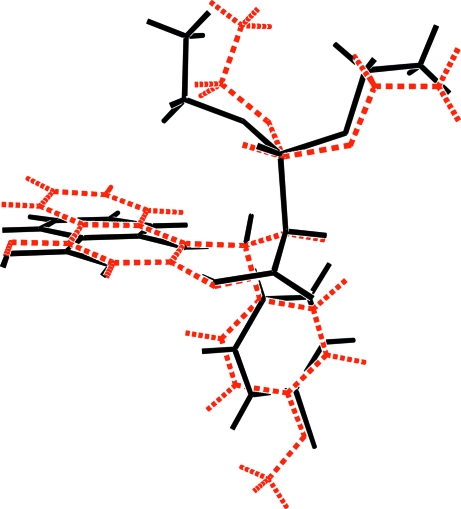
A superposition of structures (I) and (II). The latter is indicated in the dashed line. The least-squares fit was based on all common non-H atoms of the 3,4-dihydrocoumarin moiety; the r.m.s. deviation was 0.10 Å.

## supplementary crystallographic information

### Comment

The 4-aryl-3,4-dihydrocoumarin (neoflavonoid) moiety is found in several natural compounds which show a wide range of biological activities. Among them, the anti-inflammatory, anti-oxidative and anti-aging properties are best recognized (Zhang *et al.*, 2006). A number of neoflavonoids isolated from various plant sources revealed cytotoxic and chemopreventive activity against cancer (Bailly *et al.*, 2003). Morever, some 4-aryl-3,4-dihydrocoumarin derivatives exhibit estrogenic activity (Roelens *et al.*, 2005). The most common method for the synthesis of 4-aryl-3,4-dihydrocoumarins involves Michael type addition of electron-rich hydroxyarenes to cinnamic acids or their derivatives (Aoki *et al.*, 2005; Li *et al.*, 2005; Rizzi *et al.*, 2006). Unfortunately this method is limited to the reactions of cinnamic acid bearing electron-donationg groups on the aromatic ring. Recently we developed a novel synthesis of the dihydrocoumarins based on CF_3_SO_3_H promoted Friedel Crafts reaction of electron-rich hydroxyarenes (Krawczyk *et al.*, 2007*a*) with the (*E*)-3-aryl-2-(diethoxyphosphoryl)acrylic acids. The title compound (I) is a key product of that synthesis.

The title compound (I) represents a novel dihydrocoumarine analog in which the δ-valerolactone ring bears the P—C bond. A related compound (II) in which the electron-donating *para*-methoxy substituent was replaced by the electron-withdrawing Br atom has been published by us recently (Krawczyk *et al.*, 2007*b*). A search of the Cambridge Structural Database (Version 5.29; Allen, 2002) showed that crystal structures of the related compounds have not been reported so far.

A view of (I), with atom numbering scheme is shown in Fig. 1. The δ-valerolactone and naphtalene moieties are almost coplanar with one another. The former ring adopts conformation close to a ^4^*S*_3_ screw-boat (Boeyens, 1978), with O1, C1, C3, C4 and C9 almost coplanar (the average r.m.s. deviation from the mean plane is 0.09 Å) and C2 situated at the flap. The Cremer & Pople (1975) puckering parameters for the ring atom sequence O1/C1/C2/C3/C4/C9 are: *Q* = 0.489 (2) Å, θ = 113.6 (2)° and φ = 327.5 (3)°. Both exocyclic substituents, namely the diethoxyphosphoryl and phenyl groups occupy axial positions in respect to the δ-valerolactone ring.

The former group adopts an almost synperiplanar conformation along the P—C2 bond (Fig. 2). This arrangement is stabilized by electrostatic interactions of the oppositely charged phosphoryl O4 (-0.70 e) with the carbonyl C1 (0.79 e) atoms [the C1···O2 distance is 2.966 (2) Å] Atomic charges derived from electrostatic potentials were calculated using *GAUSSIAN03* (Frisch *et al.*, 2004) at the MP2/6-311++G(d,p) level for the X-ray determined coordinates. Grid points were selected according to the CHELPG procedure of Breneman & Wiberg (1990).

The phosphorus atom is located within the center of distorted tetrahedron with valency angles ranging in value from 99.90 (9) to 115.74 (11)°. On average the O—P—O type angles [111.6 (2)°] are larger then O—P—C [106.9 (2)°]. This is a general feature, often encountered in phosphorus compounds, indicating a significance of repulsive Coulombic type interactions between the oxygen atoms bearing the negative charge (Gillespie & Popelier, 2001).

A superposition of structures (I) and (II), as presented in Fig. 3, clearly shows similarity of the neoflavonoid fragments. The major difference is position of the O5—C22—C23 diethoxy group. In (I) this group points away from the naphtalene fragment while in (II) it is almost paralel and involved in the C—H—π(arom) interaction. The latter stabilizes a virtual eclipsed conformation along the axial P—C bond as was found in (II).

In the crystal molecules form centrosymmetric dimers connected by strong, practically linear hydrogen bonds linking phosphoryl and hydroxyl groups of both monomers. In terms of Etter's graph-set terminology (Etter, 1990; Bernstein, *et al.*, 1995) this system can be described as *R*^2^_2_(20).

### Experimental

The solution 1,3-dihydroxynaphtalene in CH_2_Cl_2_, trifluoromethanesulfonic acid and (*E*)-2-diethoxyphosphoryl-3-(4-methoxyphenyl)acrylic acid were added and a resulting mixture was left at room temperature for 1 day. After the acrylic acid was completely reacted, saturated NaHCO_3_ solution was added. The organic layer was separated, washed with water and dried over MgSO_4_. Evaporation of the solvent under reduced preasure gave a crude product which was purified by column chromatography and recrystalized from diethyl ether.

### Refinement

H atoms were located on difference Fourier maps and refined as riding on their carrier O or C atoms with *U*_iso_(H) = 1.2*U*_eq_(O or C). The methyl groups were allowed to rotate about their local threefold axis. AFIX 84, AFIX 14, AFIX 24, AFIX 44 and AFIX 138 procedures as in *SHELXTL* (Sheldrick, 2008) were applied.

### Crystal data

**Table:**

C_24_H_25_O_7_P	*D*_x_ = 1.348 Mg m^−^^3^
*M**_r_* = 456.41	Melting point = 396–398 K
Monoclinic, *C*2/*c*	Mo *K*α radiation λ = 0.71073 Å
*a* = 21.6231 (17) Å	Cell parameters from 8721 reflections
*b* = 10.0018 (8) Å	θ = 2.2–23.3º
*c* = 22.4011 (17) Å	µ = 0.17 mm^−^^1^
β = 111.8060 (10)º	*T* = 293 (2) K
*V* = 4498.0 (6) Å^3^	Prism, colourless
*Z* = 8	0.30 × 0.20 × 0.15 mm
*F*_000_ = 1920	

### Data collection

**Table:**

Bruker SMART APEX diffractometer	5076 independent reflections
Radiation source: fine-focus sealed tube	3585 reflections with *I* > 2σ(*I*)
Monochromator: graphite	*R*_int_ = 0.044
*T* = 293(2) K	θ_max_ = 27.4º
ω scans	θ_min_ = 2.0º
Absorption correction: multi-scan(SADABS; Bruker, 2003)	*h* = −27→27
*T*_min_ = 0.951, *T*_max_ = 0.976	*k* = −12→12
37601 measured reflections	*l* = −28→28

### Refinement

**Table:**

Refinement on *F*^2^	Secondary atom site location: difference Fourier map
Least-squares matrix: full	Hydrogen site location: difference Fourier map
*R*[*F*^2^ > 2σ(*F*^2^)] = 0.045	H-atom parameters constrained
*wR*(*F*^2^) = 0.137	*w* = 1/[σ^2^(*F*_o_^2^) + (0.0691*P*)^2^ + 2.0425*P*] where *P* = (*F*_o_^2^ + 2*F*_c_^2^)/3
*S* = 1.05	(Δ/σ)_max_ = 0.001
5076 reflections	Δρ_max_ = 0.30 e Å^−^^3^
310 parameters	Δρ_min_ = −0.36 e Å^−^^3^
Primary atom site location: structure-invariant direct methods	Extinction correction: none

### Special details

**Table:**

Geometry. All e.s.d.'s (except the e.s.d. in the dihedral angle between two l.s. planes) are estimated using the full covariance matrix. The cell e.s.d.'s are taken into account individually in the estimation of e.s.d.'s in distances, angles and torsion angles; correlations between e.s.d.'s in cell parameters are only used when they are defined by crystal symmetry. An approximate (isotropic) treatment of cell e.s.d.'s is used for estimating e.s.d.'s involving l.s. planes.
Refinement. Refinement of *F*^2^ against ALL reflections. The weighted *R*-factor *wR* and goodness of fit *S* are based on *F*^2^, conventional *R*-factors *R* are based on *F*, with *F* set to zero for negative *F*^2^. The threshold expression of *F*^2^ > σ(*F*^2^) is used only for calculating *R*-factors(gt) *etc*. and is not relevant to the choice of reflections for refinement. *R*-factors based on *F*^2^ are statistically about twice as large as those based on *F*, and *R*- factors based on ALL data will be even larger.

### Fractional atomic coordinates and isotropic or equivalent isotropic displacement parameters (Å^2^)

**Table:**

	*x*	*y*	*z*	*U*_iso_*/*U*_eq_	
P	0.85826 (2)	0.18689 (5)	0.93687 (3)	0.04719 (16)	
O1	0.86909 (7)	0.50338 (13)	0.99316 (6)	0.0501 (3)	
O2	0.82123 (8)	0.38041 (16)	1.04447 (7)	0.0612 (4)	
O3	0.82989 (9)	0.07064 (16)	0.96515 (11)	0.0843 (6)	
O4	0.92748 (7)	0.22132 (16)	0.97264 (8)	0.0645 (4)	
O5	0.84281 (8)	0.14572 (19)	0.86632 (8)	0.0751 (5)	
O6	1.00680 (7)	0.72224 (17)	0.90165 (8)	0.0694 (5)	
O7	0.54354 (8)	0.70103 (17)	0.84382 (11)	0.0838 (6)	
C1	0.82904 (10)	0.40053 (19)	0.99520 (10)	0.0464 (4)	
C2	0.79907 (9)	0.31862 (18)	0.93467 (9)	0.0445 (4)	
C3	0.77848 (9)	0.40694 (18)	0.87347 (9)	0.0433 (4)	
C4	0.83847 (9)	0.49031 (17)	0.87772 (9)	0.0411 (4)	
C5	0.85316 (9)	0.53228 (18)	0.82352 (9)	0.0432 (4)	
C6	0.91093 (9)	0.60975 (19)	0.83255 (9)	0.0457 (4)	
C7	0.95280 (9)	0.6475 (2)	0.89610 (10)	0.0492 (5)	
C8	0.93774 (10)	0.60900 (19)	0.94736 (10)	0.0484 (5)	
C9	0.88100 (9)	0.53133 (18)	0.93693 (9)	0.0435 (4)	
C10	0.81079 (11)	0.5026 (2)	0.75920 (10)	0.0540 (5)	
C11	0.82642 (13)	0.5444 (2)	0.70810 (11)	0.0616 (6)	
C12	0.88486 (13)	0.6152 (2)	0.71814 (12)	0.0636 (6)	
C13	0.92573 (12)	0.6490 (2)	0.77862 (11)	0.0567 (5)	
C14	0.71632 (9)	0.48976 (18)	0.86470 (9)	0.0436 (4)	
C15	0.65938 (10)	0.4277 (2)	0.86667 (12)	0.0596 (6)	
C16	0.60208 (11)	0.4993 (2)	0.85812 (13)	0.0667 (6)	
C17	0.60088 (11)	0.6368 (2)	0.84824 (11)	0.0589 (5)	
C18	0.65601 (11)	0.6988 (2)	0.84519 (11)	0.0586 (5)	
C19	0.71301 (11)	0.6250 (2)	0.85320 (10)	0.0525 (5)	
C20	0.85865 (19)	−0.0622 (3)	0.97519 (18)	0.0957 (10)	
C21	0.80938 (19)	−0.1530 (3)	0.98260 (16)	0.0963 (10)	
C22	0.89018 (18)	0.1465 (3)	0.83444 (15)	0.0930 (9)	
C23	0.90252 (16)	0.0103 (4)	0.81830 (16)	0.0989 (10)	
C24	0.54361 (16)	0.8430 (3)	0.84361 (19)	0.1038 (12)	
H6	1.0276	0.7404	0.9401	0.083*	
H21	0.7610	0.2782	0.9357	0.053*	
H31	0.7679	0.3463	0.8355	0.049*	
H81	0.9632	0.6325	0.9862	0.058*	
H101	0.7706	0.4525	0.7514	0.065*	
H111	0.7983	0.5257	0.6674	0.074*	
H121	0.8960	0.6395	0.6832	0.076*	
H131	0.9646	0.6994	0.7848	0.068*	
H151	0.6601	0.3230	0.8750	0.071*	
H161	0.5596	0.4502	0.8591	0.080*	
H181	0.6553	0.7910	0.8377	0.070*	
H191	0.7487	0.6669	0.8508	0.063*	
H201	0.9030	−0.0639	1.0173	0.115*	
H202	0.8708	−0.0913	0.9351	0.115*	
H211	0.7686	−0.1586	0.9396	0.116*	
H212	0.7941	−0.1177	1.0185	0.116*	
H213	0.8303	−0.2473	0.9950	0.116*	
H221	0.9333	0.1881	0.8636	0.112*	
H222	0.8721	0.2019	0.7940	0.112*	
H231	0.8583	−0.0365	0.7950	0.119*	
H232	0.9286	−0.0405	0.8593	0.119*	
H233	0.9293	0.0130	0.7894	0.119*	
H241	0.5474	0.8736	0.8060	0.125*	
H242	0.5793	0.8740	0.8787	0.125*	
H243	0.5042	0.8741	0.8460	0.125*	

### Atomic displacement parameters (Å^2^)

**Table:**

	*U*^11^	*U*^22^	*U*^33^	*U*^12^	*U*^13^	*U*^23^
P	0.0439 (3)	0.0381 (3)	0.0599 (3)	−0.0019 (2)	0.0197 (2)	−0.0012 (2)
O1	0.0655 (8)	0.0447 (7)	0.0433 (7)	−0.0119 (6)	0.0239 (6)	−0.0030 (6)
O2	0.0725 (10)	0.0645 (9)	0.0561 (9)	−0.0060 (7)	0.0350 (8)	0.0033 (7)
O3	0.0837 (12)	0.0446 (9)	0.1457 (18)	0.0093 (8)	0.0673 (12)	0.0225 (10)
O4	0.0446 (8)	0.0607 (9)	0.0785 (11)	−0.0006 (7)	0.0117 (7)	−0.0075 (8)
O5	0.0616 (9)	0.0879 (12)	0.0692 (11)	0.0157 (8)	0.0167 (8)	−0.0224 (9)
O6	0.0543 (9)	0.0794 (11)	0.0696 (10)	−0.0216 (8)	0.0174 (7)	0.0139 (8)
O7	0.0596 (10)	0.0626 (11)	0.1338 (17)	0.0164 (8)	0.0414 (10)	0.0119 (10)
C1	0.0509 (10)	0.0410 (9)	0.0514 (11)	0.0028 (8)	0.0236 (9)	0.0050 (8)
C2	0.0418 (9)	0.0380 (9)	0.0562 (11)	−0.0040 (8)	0.0213 (8)	0.0010 (8)
C3	0.0451 (10)	0.0381 (9)	0.0464 (10)	−0.0037 (7)	0.0169 (8)	−0.0037 (8)
C4	0.0412 (9)	0.0383 (9)	0.0445 (10)	−0.0008 (7)	0.0170 (8)	0.0002 (7)
C5	0.0448 (10)	0.0414 (9)	0.0437 (10)	0.0087 (8)	0.0169 (8)	0.0033 (8)
C6	0.0461 (10)	0.0426 (10)	0.0511 (11)	0.0087 (8)	0.0214 (8)	0.0098 (8)
C7	0.0418 (10)	0.0459 (10)	0.0584 (12)	−0.0003 (8)	0.0169 (9)	0.0101 (9)
C8	0.0493 (11)	0.0459 (10)	0.0443 (11)	−0.0060 (8)	0.0106 (8)	0.0032 (8)
C9	0.0514 (10)	0.0378 (9)	0.0433 (10)	−0.0006 (8)	0.0198 (8)	0.0018 (7)
C10	0.0553 (12)	0.0562 (12)	0.0483 (12)	0.0062 (9)	0.0167 (9)	−0.0012 (9)
C11	0.0753 (15)	0.0636 (14)	0.0431 (11)	0.0190 (11)	0.0189 (10)	0.0050 (10)
C12	0.0793 (16)	0.0655 (14)	0.0563 (13)	0.0187 (12)	0.0371 (12)	0.0177 (11)
C13	0.0600 (12)	0.0579 (12)	0.0601 (14)	0.0096 (10)	0.0316 (11)	0.0160 (10)
C14	0.0445 (10)	0.0403 (9)	0.0432 (10)	−0.0030 (8)	0.0129 (8)	−0.0045 (8)
C15	0.0497 (12)	0.0424 (11)	0.0826 (16)	−0.0040 (9)	0.0200 (11)	−0.0020 (10)
C16	0.0469 (12)	0.0520 (12)	0.0978 (19)	−0.0041 (10)	0.0231 (12)	−0.0002 (12)
C17	0.0513 (12)	0.0538 (12)	0.0698 (14)	0.0071 (9)	0.0204 (10)	0.0034 (10)
C18	0.0631 (13)	0.0425 (11)	0.0710 (14)	0.0055 (9)	0.0262 (11)	0.0090 (10)
C19	0.0526 (11)	0.0445 (10)	0.0634 (13)	−0.0034 (9)	0.0248 (10)	0.0045 (9)
C20	0.134 (3)	0.0501 (14)	0.134 (3)	0.0191 (15)	0.085 (2)	0.0209 (15)
C21	0.150 (3)	0.0501 (14)	0.093 (2)	−0.0089 (17)	0.050 (2)	−0.0010 (14)
C22	0.128 (3)	0.091 (2)	0.0795 (19)	0.0193 (19)	0.0617 (19)	0.0055 (16)
C23	0.093 (2)	0.109 (2)	0.102 (2)	0.0270 (19)	0.0449 (18)	−0.0189 (19)
C24	0.084 (2)	0.0640 (17)	0.169 (4)	0.0255 (15)	0.053 (2)	0.0175 (19)

### Geometric parameters (Å, °)

**Table:**

P—O4	1.4524 (15)	C11—C12	1.392 (3)
P—O5	1.5449 (17)	C11—H111	0.9082
P—O3	1.5551 (16)	C12—C13	1.357 (3)
P—C2	1.8248 (19)	C12—H121	0.9322
O1—C1	1.356 (2)	C13—H131	0.9462
O1—C9	1.404 (2)	C14—C19	1.374 (3)
O2—C1	1.194 (2)	C14—C15	1.394 (3)
O3—C20	1.449 (3)	C15—C16	1.381 (3)
O5—C22	1.450 (3)	C15—H151	1.0672
O6—C7	1.352 (2)	C16—C17	1.391 (3)
O6—H6	0.8309	C16—H161	1.0483
O7—C17	1.367 (3)	C17—C18	1.368 (3)
O7—C24	1.420 (3)	C18—C19	1.390 (3)
C1—C2	1.509 (3)	C18—H181	0.9346
C2—C3	1.551 (3)	C19—H191	0.8967
C2—H21	0.9241	C20—C21	1.455 (4)
C3—C4	1.515 (2)	C20—H201	1.0668
C3—C14	1.528 (3)	C20—H202	1.0668
C3—H31	1.0001	C21—H211	1.0385
C4—C9	1.366 (3)	C21—H212	1.0385
C4—C5	1.428 (3)	C21—H213	1.0385
C5—C6	1.419 (3)	C22—C23	1.459 (4)
C5—C10	1.422 (3)	C22—H221	1.0092
C6—C13	1.415 (3)	C22—H222	1.0092
C6—C7	1.425 (3)	C23—H231	1.0166
C7—C8	1.360 (3)	C23—H232	1.0166
C8—C9	1.397 (3)	C23—H233	1.0166
C8—H81	0.8711	C24—H241	0.9270
C10—C11	1.374 (3)	C24—H242	0.9270
C10—H101	0.9613	C24—H243	0.9270
			
O4—P—O5	114.48 (10)	C11—C12—H121	119.9
O4—P—O3	115.74 (11)	C12—C13—C6	120.9 (2)
O5—P—O3	104.43 (11)	C12—C13—H131	119.6
O4—P—C2	114.36 (9)	C6—C13—H131	119.6
O5—P—C2	106.37 (9)	C19—C14—C15	117.34 (18)
O3—P—C2	99.90 (9)	C19—C14—C3	122.67 (17)
C1—O1—C9	120.89 (15)	C15—C14—C3	119.98 (17)
C20—O3—P	122.52 (17)	C16—C15—C14	121.5 (2)
C22—O5—P	125.35 (18)	C16—C15—H151	119.3
C7—O6—H6	109.5	C14—C15—H151	119.3
C17—O7—C24	117.9 (2)	C15—C16—C17	119.8 (2)
O2—C1—O1	118.05 (18)	C15—C16—H161	120.1
O2—C1—C2	125.32 (18)	C17—C16—H161	120.1
O1—C1—C2	116.61 (16)	O7—C17—C18	124.5 (2)
C1—C2—C3	111.81 (15)	O7—C17—C16	116.0 (2)
C1—C2—P	107.75 (13)	C18—C17—C16	119.4 (2)
C3—C2—P	113.78 (13)	C17—C18—C19	120.0 (2)
C1—C2—H21	107.8	C17—C18—H181	120.0
C3—C2—H21	107.8	C19—C18—H181	120.0
P—C2—H21	107.8	C14—C19—C18	121.9 (2)
C4—C3—C14	113.67 (15)	C14—C19—H191	119.0
C4—C3—C2	107.47 (15)	C18—C19—H191	119.0
C14—C3—C2	111.86 (15)	O3—C20—C21	107.4 (3)
C4—C3—H31	107.9	O3—C20—H201	110.2
C14—C3—H31	107.9	C21—C20—H201	110.2
C2—C3—H31	107.9	O3—C20—H202	110.2
C9—C4—C5	117.07 (17)	C21—C20—H202	110.2
C9—C4—C3	118.56 (16)	H201—C20—H202	108.5
C5—C4—C3	124.35 (16)	C20—C21—H211	109.5
C6—C5—C10	117.40 (18)	C20—C21—H212	109.5
C6—C5—C4	120.10 (17)	H211—C21—H212	109.5
C10—C5—C4	122.48 (18)	C20—C21—H213	109.5
C13—C6—C5	119.73 (19)	H211—C21—H213	109.5
C13—C6—C7	121.19 (19)	H212—C21—H213	109.5
C5—C6—C7	119.08 (17)	O5—C22—C23	110.2 (3)
O6—C7—C8	123.23 (19)	O5—C22—H221	109.6
O6—C7—C6	116.41 (18)	C23—C22—H221	109.6
C8—C7—C6	120.37 (18)	O5—C22—H222	109.6
C7—C8—C9	119.15 (19)	C23—C22—H222	109.6
C7—C8—H81	120.4	H221—C22—H222	108.1
C9—C8—H81	120.4	C22—C23—H231	109.5
C4—C9—C8	124.20 (17)	C22—C23—H232	109.5
C4—C9—O1	121.96 (17)	H231—C23—H232	109.5
C8—C9—O1	113.77 (16)	C22—C23—H233	109.5
C11—C10—C5	121.0 (2)	H231—C23—H233	109.5
C11—C10—H101	119.5	H232—C23—H233	109.5
C5—C10—H101	119.5	O7—C24—H241	109.5
C10—C11—C12	120.7 (2)	O7—C24—H242	109.5
C10—C11—H111	119.7	H241—C24—H242	109.5
C12—C11—H111	119.7	O7—C24—H243	109.5
C13—C12—C11	120.2 (2)	H241—C24—H243	109.5
C13—C12—H121	119.9	H242—C24—H243	109.5
			
O4—P—C2—C1	−28.73 (16)	C13—C6—C7—C8	179.94 (18)
O5—P—C2—C3	−31.54 (16)	C5—C6—C7—C8	0.4 (3)
O4—P—O3—C20	−58.5 (3)	O6—C7—C8—C9	−179.72 (18)
O5—P—O3—C20	68.3 (3)	C6—C7—C8—C9	0.4 (3)
C2—P—O3—C20	178.2 (2)	C5—C4—C9—C8	−1.1 (3)
O4—P—O5—C22	−3.4 (3)	C3—C4—C9—C8	−179.64 (17)
O3—P—O5—C22	−131.0 (2)	C5—C4—C9—O1	175.78 (15)
C2—P—O5—C22	123.9 (2)	C3—C4—C9—O1	−2.8 (3)
C9—O1—C1—O2	179.21 (17)	C7—C8—C9—C4	−0.1 (3)
C9—O1—C1—C2	0.5 (2)	C7—C8—C9—O1	−177.13 (17)
O2—C1—C2—C3	143.09 (19)	C1—O1—C9—C4	22.1 (3)
O1—C1—C2—C3	−38.3 (2)	C1—O1—C9—C8	−160.77 (17)
O2—C1—C2—P	−91.2 (2)	C6—C5—C10—C11	1.8 (3)
O1—C1—C2—P	87.41 (17)	C4—C5—C10—C11	−179.95 (18)
O5—P—C2—C1	−156.12 (13)	C5—C10—C11—C12	1.1 (3)
O3—P—C2—C1	95.53 (15)	C10—C11—C12—C13	−3.0 (3)
O4—P—C2—C3	95.84 (15)	C11—C12—C13—C6	1.9 (3)
O5—P—C2—C3	−31.54 (16)	C5—C6—C13—C12	1.0 (3)
O3—P—C2—C3	−139.90 (15)	C7—C6—C13—C12	−178.5 (2)
C1—C2—C3—C4	52.53 (19)	C4—C3—C14—C19	8.2 (3)
P—C2—C3—C4	−69.83 (17)	C2—C3—C14—C19	130.13 (19)
C1—C2—C3—C14	−72.89 (19)	C4—C3—C14—C15	−173.41 (18)
P—C2—C3—C14	164.74 (12)	C2—C3—C14—C15	−51.5 (2)
C14—C3—C4—C9	90.9 (2)	C19—C14—C15—C16	−0.7 (3)
C2—C3—C4—C9	−33.4 (2)	C3—C14—C15—C16	−179.2 (2)
C14—C3—C4—C5	−87.6 (2)	C14—C15—C16—C17	−1.0 (4)
C2—C3—C4—C5	148.11 (17)	C24—O7—C17—C18	−7.7 (4)
C9—C4—C5—C6	1.9 (3)	C24—O7—C17—C16	170.6 (3)
C3—C4—C5—C6	−179.65 (16)	C15—C16—C17—O7	−176.4 (2)
C9—C4—C5—C10	−176.39 (17)	C15—C16—C17—C18	2.0 (4)
C3—C4—C5—C10	2.1 (3)	O7—C17—C18—C19	177.0 (2)
C10—C5—C6—C13	−2.8 (3)	C16—C17—C18—C19	−1.3 (4)
C4—C5—C6—C13	178.90 (17)	C15—C14—C19—C18	1.4 (3)
C10—C5—C6—C7	176.76 (17)	C3—C14—C19—C18	179.84 (19)
C4—C5—C6—C7	−1.6 (3)	C17—C18—C19—C14	−0.4 (3)
C13—C6—C7—O6	0.0 (3)	P—O3—C20—C21	−162.7 (2)
C5—C6—C7—O6	−179.46 (17)	P—O5—C22—C23	114.1 (3)

### Hydrogen-bond geometry (Å, °)

**Table:**

*D*—H···*A*	*D*—H	H···*A*	*D*···*A*	*D*—H···*A*
O6—H6···O4^i^	0.83	1.87	2.701 (2)	178

Symmetry codes: (i) −*x*+2, −*y*+1, −*z*+2.
